# Exogenous Testosterone and Estrogen Blocker-Associated Venous Thrombosis Presenting With Acute Aphasia: A Case Report

**DOI:** 10.7759/cureus.90833

**Published:** 2025-08-23

**Authors:** Brandon Weissman, Shafayath Chowdhury, Sera Saju, Michael Pusatier

**Affiliations:** 1 Otolaryngology, Lake Erie College of Osteopathic Medicine, Elmira, USA; 2 Anesthesiology and Critical Care, Lake Erie College of Osteopathic Medicine, Elmira, USA; 3 Neurology, Lake Erie College of Osteopathic Medicine, Elmira, USA; 4 Family Medicine, Buffalo Medical Group, Williamsville, USA

**Keywords:** aphasia, cerebral vein thrombosis (cvt), hypercoagulable, hypoplastic v4, testosterone

## Abstract

Cerebral venous thrombosis (CVT) is an uncommon cause of stroke that often presents with vague or subacute neurological symptoms that make timely diagnosis challenging. We describe the case of a 52-year-old male patient who presented with an acute onset of aphasia, a left-sided headache, and visual field deficits. Initial non-contrast computed tomography (CT) imaging was unremarkable, but subsequent magnetic resonance venography (MRV) confirmed a thrombosis in the left transverse and sigmoid sinuses. The patient had been started on testosterone replacement therapy (TRT) and estrogen blockers six weeks prior to symptom onset. Laboratory studies revealed elevated hemoglobin, hematocrit, and testosterone levels, which were suggestive of erythrocytosis and increased blood viscosity. Imaging also revealed congenital hypoplasia of the V4 segment of the left vertebral artery. This variant may have contributed to altered cerebral hemodynamics. The patient was treated initially with intravenous heparin and transitioned to apixaban, a direct oral anticoagulant (DOAC). The outcome was favorable, with a full resolution of symptoms just three days after admission. This case highlights the importance of maintaining a high clinical suspicion for CVT in patients with non-specific neurological complaints, especially when prothrombotic risk factors such as recent hormone therapy are present. It also underscores the diagnostic value of contrast-enhanced imaging modalities and supports the use of DOACs as an effective treatment option for CVT.

## Introduction

Cerebral venous thrombosis (CVT) is a rare condition in which a clot forms in the cerebral venous system and obstructs outflow. This condition affects three in 100,000 people per year [1]. CVT occurs 62% of the time in the superior sagittal sinus, 41% to 45% in the transverse sinus, 18% in the straight sinus, 17.1% in the cortical veins, 12% in the jugular veins, and 11% in the vein of Galen or internal cerebral vein [2]. The cerebral venous system is characterized by two subsystems: the superficial and deep venous systems. The superficial cerebral system comprises valveless cortical veins that empty into the superior sagittal, transverse, sigmoid, and cavernous dural sinuses. In contrast, the deep system begins with the internal cerebral and basal veins, which join to form the vein of Galen. This system then drains through the straight sinus to the confluence of sinuses before reaching the transverse-sigmoid pathway [3].

CVT is thought to increase pressure in the venous system, which decreases capillary perfusion pressure, leading to an increase in cerebral blood volume and ultimately causing intracranial hypertension. Within the venous sinus system, collateral circulation exists; however, intracranial hypertension can still compromise the blood-brain barrier and potentially lead to vasogenic edema [2]. The risk for developing CVT is based on risk factors, and 85% of affected patients will have at least one risk factor. These risk factors induce prothrombotic conditions, such as the factor V Leiden mutation, infections, trauma, vasculitis, intracranial pathology (including tumors or arteriovenous malformations), hematological issues, myeloproliferative disorders, systemic diseases, and certain medications, including hormone therapy [3]. Patients will often present with a variable presentation where symptoms can manifest in an acute, subacute, or chronic fashion. The most common presenting symptom is headache, which typically increases in severity over several days to weeks. Increased intracranial pressure will lead to papilledema and diplopia from an abducens nerve palsy. Neurological signs can also be observed, including motor weakness, hemiparesis, and seizures [4]. Given the variety of presenting symptoms, clinicians should have a high index of suspicion to identify CVT. A non-contrast computed tomography (CT) scan should be obtained first. This should be followed by a CT venography and a magnetic resonance imaging (MRI) scan with magnetic resonance venography (MRV), which is widely considered the gold standard in diagnosis. If the diagnosis is not finalized after these imaging modalities, cerebral angiography is indicated [4].

Treatment of CVT primarily relies on anticoagulant therapy. Even when hemorrhage is present, the American Heart Association and American Stroke Association recommend anticoagulation as the ideal therapeutic regimen [5]. There are still ongoing discussions about the choice of anticoagulant therapy in these cases, with some studies suggesting the use of low-molecular-weight heparin; however, unfractionated heparin remains the more common therapy of choice [5]. If anticoagulation is contraindicated, endovascular thrombolysis can be considered as an alternative. Following initial anticoagulation, long-term anticoagulation should be initiated for three to 12 months [5]. Most patients will fully recover from CVT, but one study found that patients will often have residual symptoms of concentration impairment, headache, depression, and fatigue [6]. We present the case of a male patient who presented to the emergency room (ER) with subacute neurologic symptoms and imaging findings that ended up being diagnostic of a CVT. 

## Case presentation

A 52-year-old male patient woke up in the middle of the night around 3:30 am and had a discussion with his wife, during which his speech and comprehension appeared normal. Subsequently, they both returned to sleep. The patient's wife called him around 6:30 am the same morning and noticed he was having trouble finding his words. The patient could not recall the names of his colleagues at that time. The patient's wife was concerned and had their son check on him. At 7 am, the son checked on the patient, and the patient could not recall the name of their dog. The patient was then brought to the ER, where he endorsed a dull and constant left-sided headache for the past three weeks. On presentation, it was discovered that the patient had gone to the urgent care one week prior, where he was diagnosed with an ear infection and initiated treatment with a ten-day course of prednisone and amoxicillin. The patient was recently started on testosterone injections for low libido six weeks before presentation. The patient also started taking estrogen blockers two weeks prior. The patient had a past medical history of essential hypertension, hyperlipidemia, and obstructive sleep apnea. On physical exam, the patient was afebrile, had a left upper quadrant visual field cut, and had a National Institutes of Health (NIH) score of two for aphasia. Initially, there was a concern for a cerebral vascular accident, so neuroimaging and labs were obtained. The lab values presented upon arrival at the ER included mild leukocytosis, erythrocytosis, and elevated markers for coagulation studies, as displayed in Table 1.

**Table 1:** The patient's laboratory values on presentation to the emergency room K/µL: thousands per microliter; g/dL: grams per deciliter; fL: femtoliters; mg/dL: milligrams per deciliter; mmol/L: millimoles per liter; APTT: activated partial thromboplastin time; PT: prothrombin time; INR: international normalized ratio; ng/dL: nanograms per deciliter; pg/mL: picograms per milliliter

Parameter	Result	Reference range
White blood count	11.1 K/uL	4.0-10.5 K/uL
Red blood count	6.39 K/uL	4.70-6.0 K/uL
Hemoglobin	18.4 g/dL	14.0-18.0 g/dL
Hematocrit	54.8%	42-52%
Mean corpuscular volume	85.8 fL	78.0-100.0 fL
Glucose level	107 mg/dL	60.0-100.0 mg/dL
Creatinine	1.33 mg/dL	0.4-1.40 mg/dL
Sodium level	140 mmol/L	135-140 mmol/L
APTT	90.5 seconds	25.0-34.0 seconds
PT	13.8 seconds	11.0-13.5 seconds
INR	1.04	0.8-1.1
Antithrombin III level	105%	80-120%
Testosterone level	790 ng/dL	193-740 ng/dL
Estradiol	92 pg/ml	10-50 pg/ml

**Figure 1:**
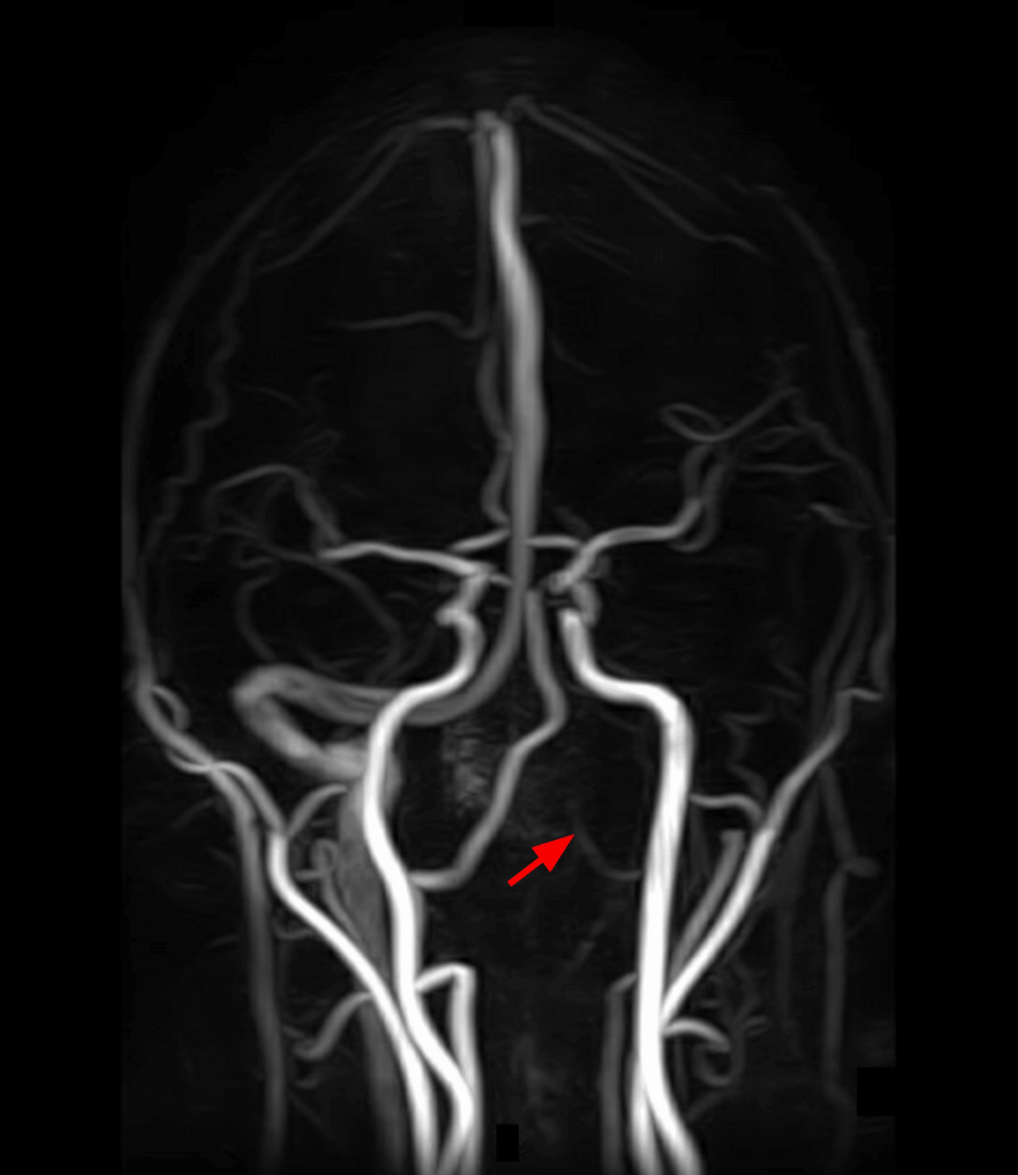
Coronal magnetic resonance angiography of the vertebrobasilar system; the red arrow depicts the hypoplastic left V4 segment of the vertebral artery.

**Figure 2:**
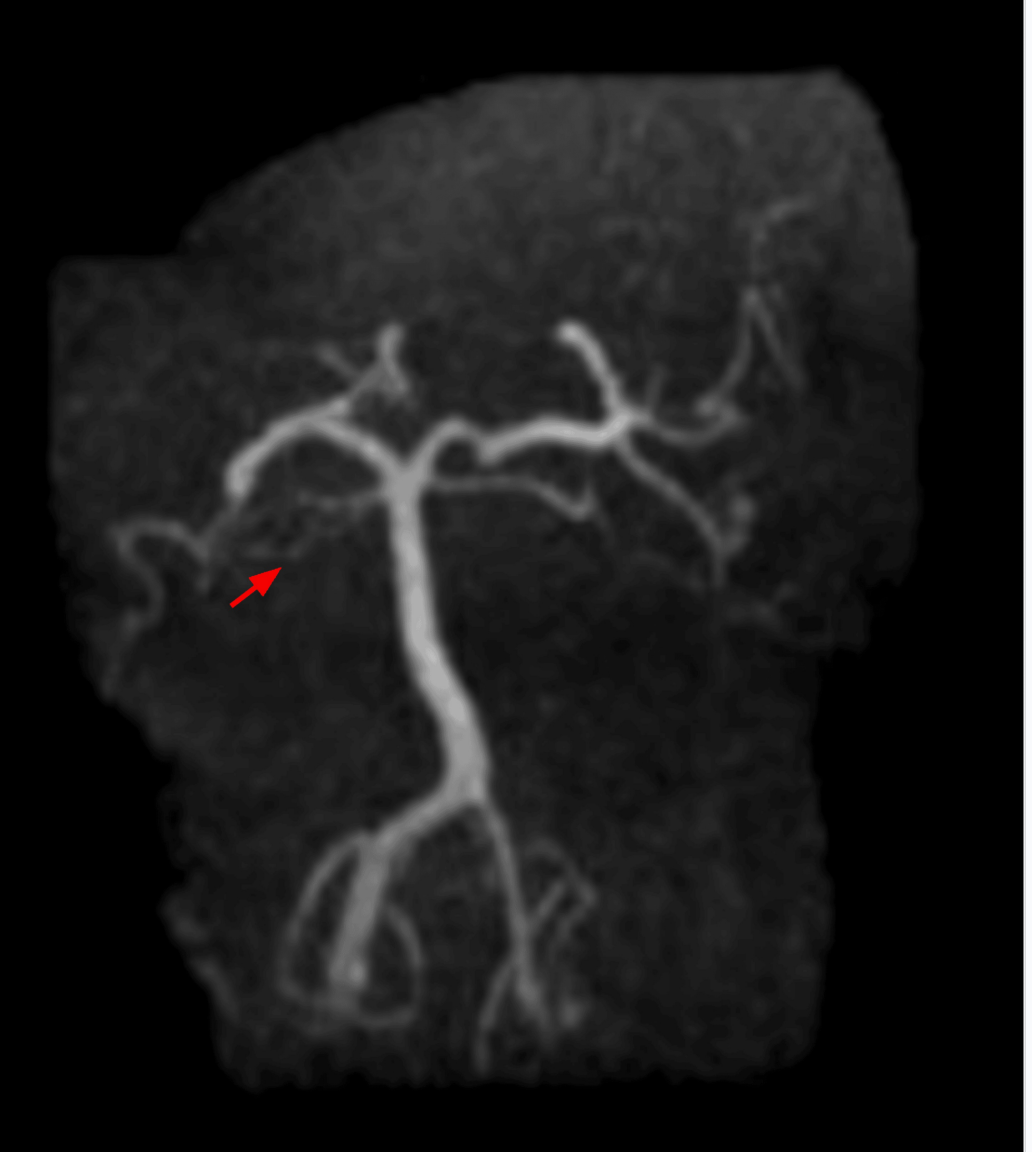
Coronal magnetic resonance angiography of the head; the red arrow indicates absent flow in the left transverse sinus consistent with cerebral venous thrombosis.

The patient denied visual disturbances, photophobia, nausea, or vomiting. A non-contrast CT scan of the head was unremarkable. However, the contrast-enhanced CT scan showed that the right transverse and sigmoid sinuses were dominant. There was found to be minimal opacification of a hypoplastic left transverse sinus medially. The distal left transverse and sigmoid sinuses were not opacified, consistent with occlusion. The findings were age-indeterminate, and an MRI was recommended. A 3D time-of-flight magnetic resonance angiography (MRA)/MRV was obtained of the brain with and without intravenous contrast, utilizing 20 cubic centimeters (cc) of intravenous Multihance. The coronal MRA of the vertebrobasilar system revealed stable patency of the intracranial arterial vessels and a hypoplastic intracranial portion of the left vertebral artery (V4), as seen in Figure 1. In addition, a coronal MRA of the head displayed diminished flow in the lateral aspect of the left transverse sinus, as seen in Figure 2. 

An MRV showed stable diminished flow in the medial aspect of the left transverse sinus. Still, the nearly absent flow in the left lateral transverse and sigmoid sinus raised concerns about thrombosis or severe sinus atresia. These findings, in conjunction, confirmed the diagnosis of left CVT in this patient. More specifically, the patient was diagnosed with left transverse/sigmoid sinus thrombosis and started on a heparin drip. He was later transitioned to a direct oral anticoagulant (DOAC). Apixaban was initiated at 10 mg twice daily for seven days, followed by 5 mg twice daily. Following admission, the patient reported resolution of headache with normalization of vision and speech after three days. Apixaban 5 mg was continued twice daily, and the patient recovered with no complications.

## Discussion

A 52-year-old male patient presented with subacute neurological symptoms, including severe aphasia, left-sided headache, and visual field deficits. Although an initial non-contrast CT did not reveal acute findings, subsequent imaging studies, as seen in Figure 1 and Figure 2, confirmed left transverse/sigmoid sinus thrombosis. The patient was also found to have elevated hematocrit levels on presentation, which predisposes to thrombotic complications due to increased blood viscosity. The clinical picture, combined with recent exogenous hormone therapy, guided the diagnostic approach toward CVT rather than a stroke or an alternative etiology.

A key component of this patient’s recent medical history was the initiation of testosterone replacement therapy (TRT) and estrogen blocker therapy approximately 6 weeks before the onset of symptoms. It is often stated that TRT can increase the risk of venous thromboembolism (VTE), particularly in the early months following initiation [7]. This occurs primarily through erythrocytosis induced by stimulation of erythropoietin, which increases blood viscosity and enhances thrombotic risk [8]. Several published case reports support this association, like where Pendyala et al. reported the case of a 49-year-old male patient with chronic testosterone use who developed CVT secondary to testosterone-induced polycythemia [9]. Another case described a 33-year-old male patient using illicit androgenic-anabolic steroids who developed extensive superior sagittal sinus thrombosis, revealing that even non-prescription androgen exposure may similarly precipitate CVT [10]. These examples support a mechanistic link between exogenous androgens and CVT.

Beyond isolated case reports, larger data sets provide a more nuanced view of this association. Meta-analyses of randomized trials and observational studies have not shown a statistically significant overall association between TRT and VTE [11]. Similarly, another study demonstrated that TRT may carry a transiently increased risk of thromboembolic and cardiovascular events, particularly in patients with coexisting risk factors [12]. When viewed in unison, these findings suggest that the absolute risk may be modest in the general population, but it is important to be vigilant during the early months of therapy, especially when additional prothrombotic factors are present.

The utility of various imaging techniques in diagnosing CVT was investigated in this case. The limited sensitivity of a non-contrast CT scan alone led to the use of contrast-enhancing imaging methods, such as MRA and MRV, which were essential in making a definitive diagnosis. The gold standard for detecting thrombi within venous sinuses is considered to be MRV [13]. A noteworthy finding from the MRA was hypoplasia of the left V4 segment of the vertebral artery. This is a congenital anatomic variant often associated with compromised posterior circulation in the brain. The identified hypoplasia can often alter cerebral hemodynamics and predispose individuals to ischemic events in the posterior circulation [14]. While congenital vascular variants can impact cerebral hemodynamics, the extent to which they contribute to CVT remains unclear and should be regarded as speculative [14]. However, the presence of this anomaly in combination with hormone-induced prothrombotic changes illustrates how multiple risk factors can converge to provoke thrombosis.

In general, the management of CVT centers on anticoagulation. International guidelines still recommend low molecular weight heparin (LMWH) followed by warfarin as the standard of care [13]. Recent studies have increasingly supported the efficacy of DOACs in the management of CVT. It was found that DOACs have comparable or even superior recanalization rates and fewer bleeding complications compared to traditional anticoagulants in cases of CVT [15]. However, these studies are limited by small sample sizes and short follow-up periods. High-quality randomized trials are needed before DOACs can be universally considered equivalent to the traditional therapy. In our patient, prompt initiation of heparin and transition to apixaban resulted in complete neurological recovery, which is consistent with emerging evidence.

## Conclusions

CVT is a condition of multifactorial etiology that presented in this patient with a recent history of exogenous hormone therapy and findings of vertebral artery hypoplasia. This is a rare but potentially severe condition that requires a high index of suspicion when patients present with atypical neurological symptoms. In this case, the simultaneous presence of TRT-induced erythrocytosis, estrogen-blocker use, and a congenital vascular anomaly created a convergence of prothrombotic risk factors not often described together in the literature. The patient experienced a rapid clinical recovery within three days of anticoagulation initiation, and follow-up at discharge demonstrated normalization of neurological function without residual deficits. Identifiable risk factors like recent hormone therapy initiation may be vital in ordering the proper diagnostic testing to include or exclude differential diagnoses effectively. Early recognition through advanced imaging techniques is crucial for identifying anatomical anomalies and ensuring a timely diagnosis, thereby facilitating the rapid initiation of anticoagulation therapy. DOACs proved to be an effective and safe therapeutic option in this case, which had a positive clinical outcome. This case uniquely highlights how overlapping acquired and congenital factors can synergistically predispose to CVT, reinforcing the importance of comprehensive history taking and tailored follow-up in patients on hormone therapy.
